# Plumbagin inhibits fungal growth, HMGB1/LOX-1 pathway and inflammatory factors in *A. fumigatus* keratitis

**DOI:** 10.3389/fmicb.2024.1383509

**Published:** 2024-04-09

**Authors:** Fan Cong, Lingwen Gu, Jing Lin, Guibo Liu, Qian Wang, Lina Zhang, Menghui Chi, Qiang Xu, Guiqiu Zhao, Cui Li

**Affiliations:** Department of Ophthalmology, The Affiliated Hospital of Qingdao University, Qingdao, Shandong, China

**Keywords:** *Aspergillus fumigatus* keratitis, plumbagin, HMGB1, LOX-1, antifungal

## Abstract

To investigate the anti-inflammatory and antifungal effects of plumbagin (PL) in *Aspergillus fumigatus* (*A. fumigatus*) keratitis, the minimum inhibitory concentration (MIC), time-killing curve, spore adhesion, crystal violet staining, calcium fluoride white staining, and Propidium Iodide (PI) staining were employed to assess the antifungal activity of PL *in vitro* against *A. fumigatus*. The cytotoxicity of PL was assessed using the Cell Counting Kit-8 (CCK8). The impact of PL on the expression of HMGB1, LOX-1, TNF-α, IL-1β, IL-6, IL-10 and ROS in *A. fumigatus* keratitis was investigated using RT-PCR, ELISA, Western blot, and Reactive oxygen species (ROS) assay. The therapeutic efficacy of PL against *A. fumigatus* keratitis was assessed through clinical scoring, plate counting, Immunofluorescence and Hematoxylin-Eosin (HE) staining. Finally, we found that PL inhibited the growth, spore adhesion, and biofilm formation of *A. fumigatus* and disrupted the integrity of its cell membrane and cell wall. PL decreased IL-6, TNF-α, and IL-1β levels while increasing IL-10 expression in fungi-infected mice corneas and peritoneal macrophages. Additionally, PL significantly attenuated the HMGB1/LOX-1 pathway while reversing the promoting effect of Boxb (an HMGB1 agonist) on HMGB1/LOX-1. Moreover, PL decreased the level of ROS. *In vivo*, clinical scores, neutrophil recruitment, and fungal burden were all significantly reduced in infected corneas treated with PL. In summary, the inflammatory process can be inhibited by PL through the regulation of the HMGB-1/LOX-1 pathway. Simultaneously, PL can exert antifungal effects by limiting fungal spore adhesion and biofilm formation, as well as causing destruction of cell membranes and walls.

## 1 Introduction

Fungal keratitis is a highly destructive infectious disease of the cornea. It occurs most commonly in the tropics and subtropics. The incidence of fungal keratitis is estimated to be over 1,000,000 cases per year, and about 10% of them will progress to corneal perforation or even enucleation (Bongomin et al., 2017; Brown et al., 2021). The disease is mainly concentrated in agricultural and outdoor workers, long-term contact lens wearers, and patients with excessive use of antibiotics and glucocorticoids (Bongomin et al., 2017). The most common pathogenic fungi are *Aspergillus* and *Fusarium* (Liu et al., 2023). In addition to the invasion and colonization of fungal hyphae in the corneal stroma, the excessive inflammation is one of the reasons for accelerating the development of fungal keratitis, which can lead to stromal damage and corneal opacity (Mills et al., 2021; Sha et al., 2021). The current treatment drugs are mainly natamycin, which is used in the form of suspension. It not only has poor dispersion and low solubility but also limits penetration into the corneal epithelium. Furthermore, natamycin is also associated with the occurrence of adverse reactions and the development of resistance (Sahay et al., 2019; Ung et al., 2019). Therefore, it is necessary to find new methods for the treatment of fungal keratitis.

Plumbagin (5-hydroxy-2-methyl-1, 4-naphthoquinone, PL) is a potent naphthoquinone compound from the roots of traditional medicinal plants. It has a variety of pharmacological properties (Jaradat et al., 2017) and has been proven to have anti-inflammatory, antimicrobial activity, anti-oxidative stress and anti-cancer effects (Pan et al., 2022; Catalani et al., 2023; Lin et al., 2023; Xiong et al., 2024). PL has been utilized in various inflammatory models, such as the lipopolysaccharide (LPS)-induced glial cell inflammation model and rheumatoid arthritis, to downregulate the expression of inflammatory factors, including TNF-α, IL-6, and IL-1β (Gupta et al., 2018; Shu et al., 2022). Moreover, in the sepsis model induced by LPS, PL has demonstrated significant efficacy in reducing HMGB1 levels and further suppressing proinflammatory cytokines (Zhang Z. et al., 2016). The accumulation of ROS in cells is a contributing factor to the development of inflammatory diseases (Kim and Kim, 2021). Multiple studies have demonstrated that PL can effectively attenuate cellular ROS levels, thereby inhibiting the release of HMGB1 and mitigating inflammatory damage (Cao et al., 2022; Xiao et al., 2022). HMGB1 is a promising and significant therapeutic target for the management of bacterial and fungal keratitis, as it plays a crucial role in influencing inflammatory factors (Wu, 2020; Yin et al., 2021). Previous research has demonstrated that HMGB1 triggers inflammation in *A. fumigatus* keratitis by upregulating LOX-1 levels, consequently elevating the levels of IL-1β, TNF-α, and other inflammatory factors (Jia-Qian Jiang et al., 2019; Wu, 2020) Therefore, PL is anticipated to have a pivotal role in treating *A. fumigatus* keratitis based on previous studies highlighting its ability to inhibit HMGB1 levels.

Furthermore, PL displays robust and extensive antifungal activity against numerous fungi, including *Fusarium graminearum* and *Candida albicans*. It not only disrupts the cell and mitochondrial membranes of the fungi, as evidenced by a concentration-dependent increase in relative electrical conductivity but also demonstrates significant anti-biofilm activity through down-regulation of FAS1 and FAS2 expression (Hassan et al., 2016; Shang et al., 2019; Qian et al., 2022; Bisso et al., 2023; Wang et al., 2023). These findings indicate that PL may also possess substantial antifungal effects in *A. fumigatus* keratitis.

The present study validated the anti-inflammatory and antifungal effects of PL against *A. fumigatus* keratitis. The findings demonstrated that PL effectively inhibited the HMGB1/LOX-1 pathway, leading to the down-regulation of proinflammatory cytokine expression and attenuating intracellular ROS levels. Moreover, PL exhibited potent inhibition of spore adhesion and biofilm formation while also disrupting fungal cell membrane and cell wall. Consequently, PL holds great promise as a highly effective therapeutic agent for the treatment of fungal keratitis.

## 2 Materials and methods

### 2.1 PL preparation

PL (HY-N1497, purity: 99.23%) was purchased from MedChemExpress (Shanghai, China). The concentration of PL was reduced to 100 mM by dissolving it in DMSO, and packaged and frozen at −80°C. The samples were taken in fractions and diluted to an appropriate concentration by phosphate-buffered saline (PBS) or corresponding cell culture medium with 1‰ DMSO.

### 2.2 *A. fumigatus* culture

A standard *A. fumigatus* strain (CPCC 3.0772), purchased from the China General Microbiological Culture Collection Center, was grown to mycelium after 2 days of cultivation in liquid Sabouraud medium at 37°C and 200 rpm. The mycelium was removed and ground in a sterile operating table, washed three times with PBS, and placed in 70% ethanol overnight to obtain inactivated mycelium, which was adjusted to a concentration of 3 × 10^8^ CFU/mL. After culturing *A. fumigatus* strains for 2–3 days at 28°C (sabouraud agar medium), conidia on the surface of the medium were collected.

### 2.3 Cell culture and *A. fumigatus* stimulation

Human corneal epithelial cells (HCECs), provided by the laboratory of Xiamen University, Fujian, China, were cultured in Dulbecco's Modified Eagle Medium (96-well plate, 37°C, 5% CO_2_) until reaching 80% confluence for CCK-8 and fungal spore adhesion experiments. RAW264.7 macrophages, purchased from the Chinese Academy of Sciences (Shanghai, China), were cultured in DMEM (light-protected 96-well plates, 37°C, 5% CO_2_) and used for CCK-8 and ROS detection when the concentration of RAW264.7 reached 80%. For the extraction of peritoneal macrophages, mice were injected intraperitoneally with 1 mL of 3% thioglycollate medium and stimulated for 7 days. When the mice were sacrificed and the abdomen was wiped with 75% alcohol, 10 mL DMEM was injected intraperitoneally and collected. After centrifugation, purification, and suspension, the cells were then suspended and plated in either 6- or 12-well plates and stimulated with inactivated hyphae for 8 or 24 h for Reverse Transcription-PCR (RT-PCR), Western Blotting (WB), and Enzyme-Linked Immunosorbent Assay (ELISA). In order to validate the HMGB1/LOX-1 pathway, peritoneal macrophages were pre-treated with Boxb for 1 h, followed by stimulation with fungi and drugs. Subsequent experiments were conducted.

### 2.4 Mice and models of fungal keratitis

Eight-week-old healthy female C57BL/6 mice were obtained from Jinan Pengyue Laboratory Animal Co., Ltd. (Jinan, China). The study was carried out following the guidelines provided by ARVO for the ethical use of animals in research related to ophthalmology and vision. Additionally, it received approval from the Ethics Committee at Qingdao University's Affiliated Hospital (QYFY WZLL 28315). After the mice were anesthetized, *A. fumigatus* spores (3 μL, 0.5 × 10^5^ μL^−1^) were injected into the corneal stroma of the right eye using a ×40 magnification stereoscopic microscope, while the left eye served as a blank control kept untreated. The experimental and control groups were treated with 4 μL PL (30 μM) and 1‰ DMSO drops on the ocular surface 4 times daily, respectively. Mouse corneas were examined daily using a slit-lamp microscope and photographed for clinical score at 1, 3, and 5 days post-infection (p.i.). After 5 days, corneas were collected for RT-PCR, ELISA, WB, and plate counting. HE staining and immunofluorescence staining were performed on infected eyeballs.

### 2.5 Cell counting kit-8 assay

After incubating HCECs with different concentrations of PL (0, 2, 3, 4, 5 and 6 μM) or 1‰ DMSO in a 96-well plate for 24 h, each well was supplemented with 10 μL of CCK-8 solution (Solarbio, Beijing, China) and further incubated for an additional period of 2 h at 37°C. The absorbance at a wavelength of 450 nm was measured using a microplate reader. RAW264.7 cells were subjected to toxicity testing using the same methodology mentioned above but with varying concentrations of PL (0, 2, 3, 4, 5, 6, 8, and 10 μM).

### 2.6 *In vivo* safety study

Chen et al. (2018) demonstrated that intravitreal injection of PL (0–50 μM) did not cause ocular toxicity in mice, which prompted this study to conduct *in vivo* toxicity tests. Mice were administered 4 μL PL (30, 40 μM) 4 times a day into the conjunctival sac of the right eye, while an equal amount of 1‰ DMSO was applied to the left eye at the same frequency. The sodium fluorescein staining of the ocular surface under cobalt blue light of the slit-lamp was observed and recorded at 1, 3, and 5 days. The ocular surface damage was assessed based on a comprehensive scoring system, which took into account corneal opacity and lesions, iris changes, as well as conjunctival congestion, bulbar chemosis, and secretion (Gomez et al., 2020; Tian et al., 2022).

### 2.7 RT-PCR assay

The RNAiso Plus reagent (Takara, Dalian, China) was utilized to extract total RNA from corneal and peritoneal macrophages in mice. Spectrophotometry was employed for the quantification of the extracted RNA. For cDNA synthesis, HiScript III RT SuperMix (Vazyme, Nanjing, China) was used to process the RNA samples. Detailed RT-PCR methods were used, as described in a previous study (Bernardino et al., 2021). Table 1 shows all primer sequences used for RT-PCR.

**Table 1:** Primer list used for RT-PCR.

**Gene (mouse)**	**Forward primer (5′-3′)**	**Reverse primer (5′-3′)**
β-actin	GATTACTGCTCTGGCTCCTAGC	GACTCATCGTACTCCTGCTTGC
IL-1β	CGCAGCAGCACATCAACAAGAGC	TGTCCTCATCCTGGAAGGTCCACG
TNF-α	ACCCTCACACTCAGATCATCTT	GGTTGTCTTTGAGATCCATGC
IL-6	CACAAGTCCGGAGAGGAGAC	CAGAATTGCCATTGCACAAC
LOX-1	AGGTCCTTGTCCACAAGACTGG	ACGCCCCTGGTCTTAAAGAATTG
IL-10	TGCTAACCGACTCCTTAATGCAGGAC	CCTTGATTTCTGGGCCATGCTTCTC
HMGB1	TGGCAAAGGCTGACAAGGCTC	GGATGCTCGCCTTTGATTTTGG

### 2.8 WB assay

The mouse corneal or peritoneal macrophages were fully lysed for 2 h using RIPA solution (Solarbio, Beijing, China) containing 1% each of protease inhibitors (PMSF) and phosphatase inhibitors. Protein concentrations were measured using the BCA kit (Solarbio). After the inclusion of SDS sample buffer (Elabscience, Wuhan, China), the samples were subjected to boiling, and protein separation was performed using a 12% SDS-PAGE gel. Subsequently, the separated proteins were transferred onto PVDF membranes (Solarbio). The membranes were then treated with a protein-free rapid-blocking buffer (1x, Yamei, Shanghai, China). Blocking was performed for 15 min, followed by overnight incubation with primary antibodies diluted in primary antibody diluent: HMGB1 antibody (1:1,000; Abcam), LOX-1 antibody (1:3,000; Proteintech), GADPH antibody (1:5,000; Elabscience), β-actin antibody (1:1,000; Elabscience). After a PBST shaken wash, the membranes were incubated with a secondary antibody diluted in secondary antibody diluent (1:5,000, Elabscience) for 60 min. Finally, the visualization of protein bands was achieved by employing an ECL chemiluminescence kit (Vazyme Biotech, Nanjing, China).

### 2.9 ELISA assay

The corneas of mice at 5 days p.i. were harvested and ground in PBS, or the cell culture medium from mouse peritoneal macrophages cultured for 24 h was collected, followed by centrifugation to obtain the supernatant. The protein levels of TNF-α, IL-6, and IL-1β were quantified using corresponding ELISA kits (BioLegend), and the absorbance was measured at 450 and 570 nm using a microplate reader.

### 2.10 Reactive oxygen species assay

The RAW264.7 cells were incubated with various concentrations of PL (0, 2, 3 μM) in a sterile and light-protected 96-well plate for 24 h. Subsequently, the cells were incubated in the dark for 30 min using the Reactive Oxygen Species Assay Kit (Beyotime). The absorbance was measured at a wavelength of 488 nm to determine ROS levels (%), which were calculated as follows: ROS level = (fluorescence value of experimental group/average fluorescence value of control group) × 100%.

### 2.11 Immunofluorescence staining

The mouse eyeballs were dissected and immersed in OCT (Sakura Tissue-Tek, Torrance, CA, USA) before being snap-frozen in liquid nitrogen. Subsequently, the corneas of the eyeballs were sectioned into 10 μm slices. These sections were then incubated with a goat anti-rat neutrophil monoclonal marker (NIMP-R14, 1:100, Santa Cruz, CA, USA) overnight. Following this primary antibody incubation step, secondary antibodies (1:500; Abcam) were applied for an additional hour. Finally, nuclear staining was performed using 4′,6-diamino-2-phenylindole dihydrochloride. Fluorescence microscopy images were captured after applying antifading reagents onto the sections.

### 2.12 HE staining

The eyeballs of mice from different experimental groups were extracted at day 5 p.i., fixed in a formalin fixative solution, embedded in paraffin, and sectioned into 5 μm slices. Subsequently, the cornea was stained with hematoxylin and eosin, followed by image acquisition.

### 2.13 MIC and time-killing curves

The broth microdilution protocol is performed in accordance with the guidelines provided by the Clinical and Laboratory Standards Institute (CLSI M27-A4) (Albuquerque et al., 2018), the PL was diluted to concentrations of 0, 1.25, 2.5, 3, 6, 8, 10, 20, 30, and 40 μM using Sand's liquid medium. The DMSO content was quantified as a concentration of 1‰. Conidia were added to achieve a uniform concentration of spore suspension at a density of 2 × 10^5^ CFU/mL. After a comprehensive blending process, the prepared mixture was subsequently transferred into a 96-well plate. The plates were then incubated for a duration ranging from 24 to 120 h and the absorbance was measured at 540 nm. Simultaneously, a control group consisting of drug-free medium without conidia was established as the negative control.

### 2.14 Anti-adhesion of *A. fumigatus* spores

In order to evaluate the anti-adhesion effect of PL on *A. fumigatus* conidia, HCECs were seeded into the four-chambered slides containing different concentrations of PL (2, 3 μM) or 1‰ DMSO, and the concentration of spores was set as 1 × 10^7^ CFU/mL, referring to previous study (Zhu et al., 2021). After a 3-h incubation period at a temperature of 37°C, the wells were rinsed with PBS in order to eliminate any non-adherent conidia. Subsequently, the cells were stained with hematoxylin and eosin, visualized under a microscope (magnification, 200×), and the average number of conidia adhered to the surface of each HCEC was calculated.

### 2.15 PI staining and calcofluor white staining

The *A. fumigatus* conidia (1 × 10^6^ CFU/mL) were incubated in 6-well plates at 37°C for 24 h, followed by additional culture in liquid medium containing various concentrations of PL (2, 3, 8, 10, 20, and 30 μM) or 1‰ DMSO for another 24 h. After being washed 3 times with sterile PBS, DNA staining agent PI (50 μg/mL; Sorobio, Beijing, China) was applied for 15 min in the dark and then examined under fluorescence microscopy (magnification, 100×; Leica, Germany). The same concentration of conidia was incubated with PL (0, 2, 3, 8, and 30 mM) or DMSO (with a concentration of 1‰) in medium for 24 h (12-well plate, 37°C). Fluorescence images were captured at the same magnification after staining with calcofluor white (CFW, 2.5 μg/mL).

### 2.16 Biofilm formation inhibition test

The biofilm formation inhibition experiment was slightly modified based on a previous study (Wang et al., 2022). In this experiment, 1 × 10^5^ CFU/mL of *A. fumigatus* conidia were co-cultured with different concentrations of PL (0, 3, 6, 8, 10, 20, and 30 μM) or 1‰ DMSO in Sargasparin medium for a duration of 48 h. After fixing the biofilm with methanol, it was stained with crystal violet (0.01%) for 15 min. Subsequently, it was washed and dried before being treated with ethanol (95%) for 5 min. The supernatant was then removed and transferred to a new 96-well plate. Finally, the absorbance at 570 nm was measured.

### 2.17 Efficacy comparison and synergy experiment between PL and natamycin

After establishing *A. fumigatus* keratitis in mice, the left eye served as a blank control was kept untreated. Four microliter PL (30 μM), 5% NATA, and PBS were, respectively, dropped on ocular surface 4 times a day. Mouse corneas were examined using a slit-lamp microscope at 1 and 5 days p.i. We employed the checkerboard method to investigate the synergistic effect of PL and NATA (Gaspar-Cordeiro et al., 2022). Different concentrations of PL and NATA were added to a 96-well plate. resulting the final drug concentrations of PL ranged from 1 to 32 μM and NATA concentrations ranged from 0 to 16 μM. The concentration of *A. fumigatus* in each well was maintained at 2 × 10^5^ CFU/mL. After incubated at 37°C for 24 h, the absorbance was measured at 540 nm, and the inhibition rate was calculated accordingly. The fractional inhibitory concentration index (FICI) was calculated at MIC_70_. FICI = (MIC_PL_ combined/MIC_PL_ alone) + (MIC_NATA_ combined/MIC_NATA_ alone). Synergism between PL and NATA was indicated when FICI ≤ 0.5. Additionally, 0.5 < FICI ≤ 1 implies additivity, 1 < FICI ≤ 2 implies indifference, and FICI > 2 implies antagonism.

### 2.18 Statistical analysis

The statistical analysis was performed using GraphPad Prism 9.5 (San Diego, CA, USA). Student's *t*-test was applied to evaluate the statistical significance between two groups. The significance among three or more groups was determined by One-way ANOVA test. All experiments were conducted in a minimum of three biological replicates and are expressed as mean ± SEM. Statistical significance was determined at *P* < 0.05.

## 3 Results

### 3.1 Evaluation of PL safety

**Figure 1:**
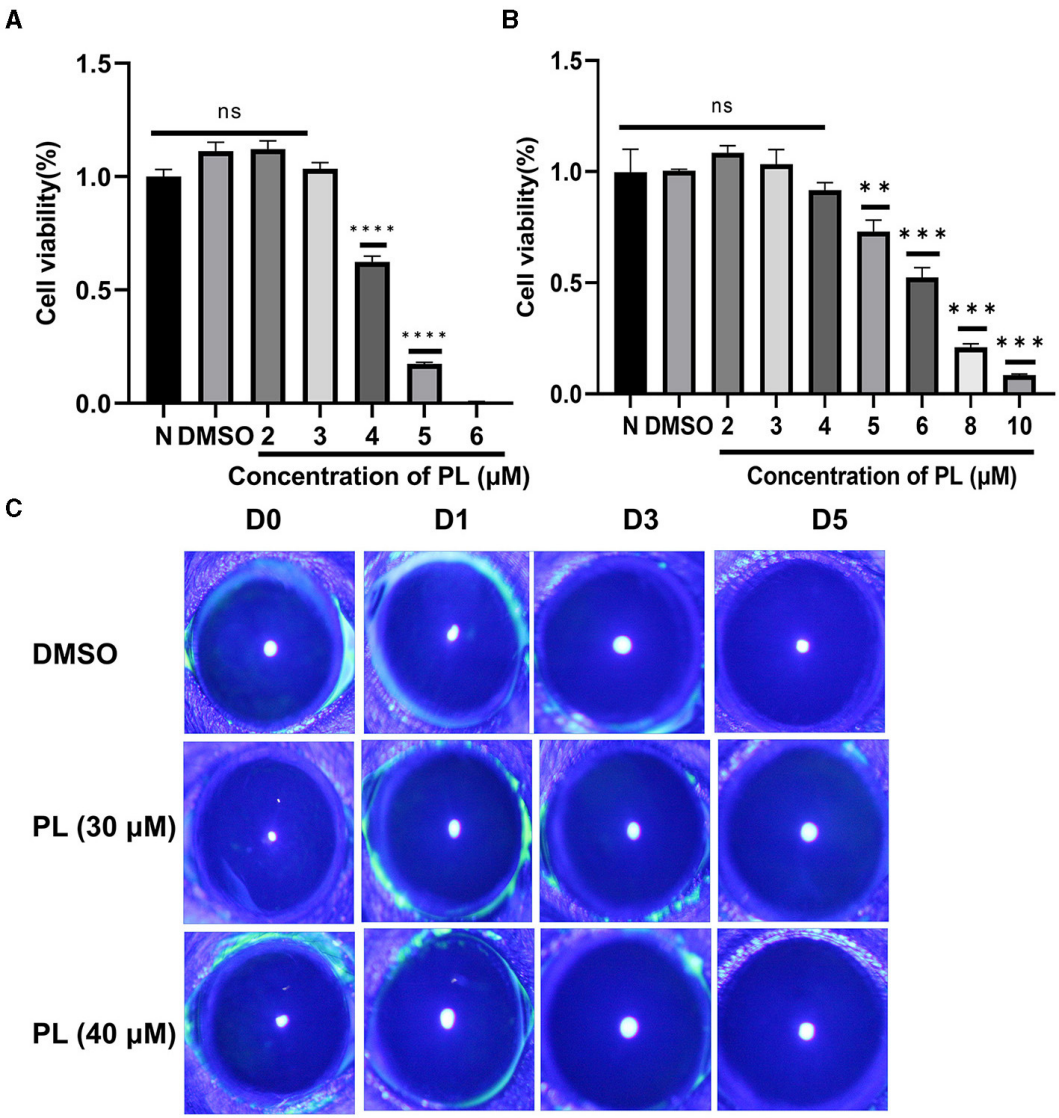
Safety evaluation of PL *in vivo* and *in vitro*. Cell viability of HCECs **(A)** treated with DMSO (1‰) and PL (2, 3, 4, 5, and 6 μM) and RAW 264.7 cells **(B)** treated with PL (2, 3, 4, 5, 6, 8, and 10 μM) for 24 h (*n* = 6/group). Corneal fluorescein staining images **(C)** were captured after 0, 1, 3, and 5 days of PL treatment. All data were displayed as mean ± SEM (ns, no significant difference, **P < 0.01, ***P < 0.001, ****P < 0.0001. N, normal cells without treatment; D, days of treatment).

To determine the appropriate concentration of PL for the treatment of fungal keratitis, experiments were carried out both *in vitro* and *in vivo*. The results from the CCK-8 assay demonstrated that PL concentrations below 3 μM exhibited no cytotoxic effects on HCECs within a 24-h timeframe (Figure 1A), while RAW264.7 cells showed no cytotoxicity at concentrations ≤ 4 μM (Figure 1B). Furthermore, the Draize test was performed to assess the potentially toxic effects of PL on mouse corneas. The findings revealed that neither 30 nor 40 μM concentrations of PL caused any damage to the mouse cornea (Figure 1C). The results suggested that 3 μM PL is safe for *in vitro* experiments, while 30 μM PL is safe for *in vivo* experiments.

### 3.2 PL alleviated the severity of mouse *A. fumigatus* keratitis

**Figure 2:**
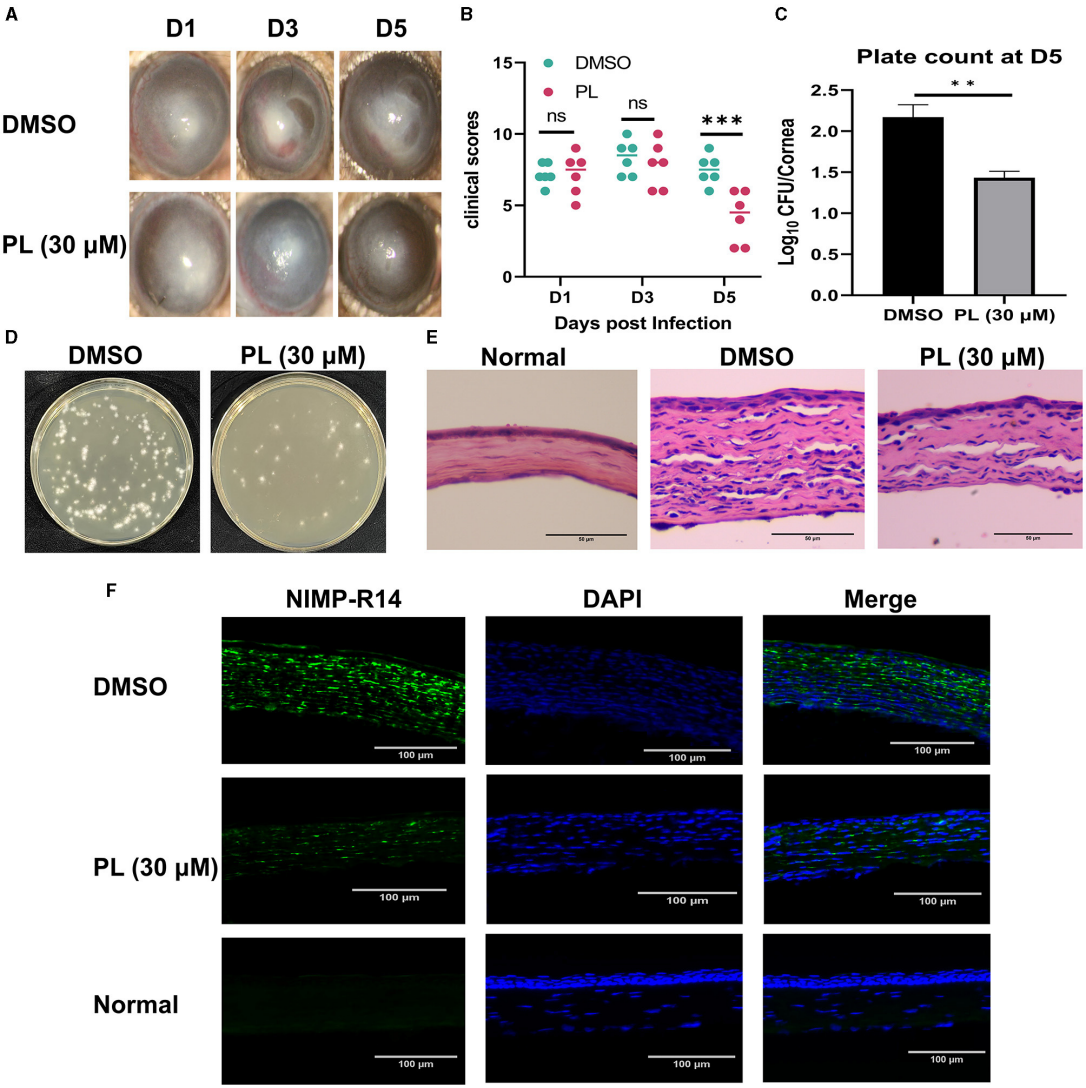
PL alleviated *A. fumigatus* keratitis in mice. Corneal changed at 1, 3, and 5 days p.i. after treatment with DMSO or PL (**A**, *n* = 6/group) and analysis of differences between the two groups **(B)**. Plate counts of fungal units **(D)** and quantities analysis **(C)** in mouse corneal homogenates at 5 days of treatment with DMSO and PL (*n* = 6/group). After 5 days of corneal infection, HE staining (**E**; Magnification, 200×; Scale bar: 50 μm) and neutrophil immunofluorescence (**F**; Magnification, 200×; Scale bar: 100 μm) were conducted on the corneas of Normal, DMSO, and PL groups. All data were displayed as mean ± SEM (ns, no significance, **P < 0.01, ***P < 0.01. D, days p.i.).

To investigate the therapeutic benefits of PL in treating keratitis caused by *A. fumigatus*, the symptomatic manifestations (including turbidity area, turbidity intensity, ulcer morphology) of mice corneas were recorded using a slit lamp at 1, 3, and 5 days p.i. (Figure 2A). Clinical scores based on symptoms were evaluated for different groups (Figure 2B), and the results demonstrated that PL treatment significantly improved symptoms and markedly reduced clinical scores compared to DMSO treatment. Plate counting experiments (Figure 2D) indicated that fungal load in the cornea was significantly lower following PL treatment than in the DMSO treatment group, with a statistically significant difference observed (Figure 2C). HE staining of corneal tissues (Figure 2E) and immunofluorescence experiment (Figure 2F) revealed that PL treatment effectively mitigated inflammatory cell infiltration and edema within the cornea. These findings suggested that PL exhibited efficacy in alleviating the inflammatory response and reducing the fungal load in the fungi-infected mouse cornea.

### 3.3 PL decreased the expression of inflammatory factors and HMGB1/LOX-1 in peritoneal macrophages, and also reduced the levels of ROS in RAW 264.7 cells

**Figure 3:**
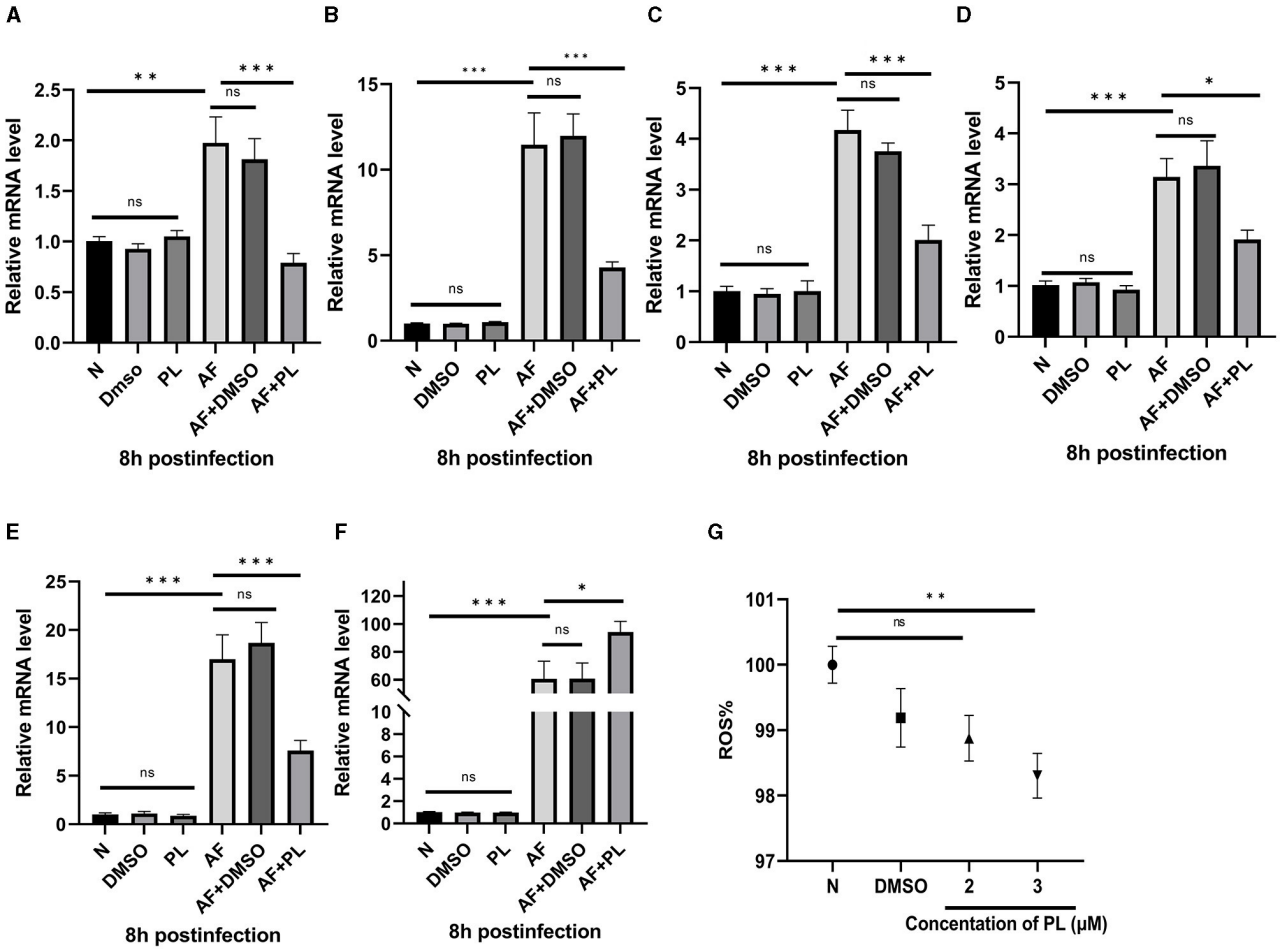
PL inhibited the level of inflammation in peritoneal macrophages *in vitro*, down-regulate the expression of HMGB1/LOX-1, and showed anti-oxidation effect. PL treatment decreased the mRNA expression of HMGB1, LOX-1, TNF-α, IL-1β, and IL-6 in peritoneal macrophages **(A–E)**, while increased the mRNA level of IL-10 **(F)**. Co-culturing PL and RAW264.7 cells for 24 h resulted in a significant reduction of intracellular ROS levels **(G)**. All data were displayed as mean ± SEM (*n* = 6/group, ns, no significance; *P < 0.05, **P < 0.01, ***P < 0.001. N, normal peritoneal macrophages or normal RAW264.7 cells; AF, *A. fumigatus*).

The anti-inflammatory effect of PL was investigated by evaluating the levels of inflammation-related factors and intracellular ROS content after treatment with PL *in vitro*. Following 8 h of stimulation with *A. fumigatus*, the mRNA levels of HMGB1 (Figure 3A), LOX-1 (Figure 3B), TNF-α (Figure 3C), IL-1β (Figure 3D), and IL-6 (Figure 3E) were significantly reduced in PL treatment group, while the mRNA expression of IL-10 (Figure 3F) was increased. Additionally, low-dose PL stimulation within a period of 24 h exhibited a concentration-dependent decrease in intracellular ROS levels (Figure 3G).

### 3.4 PL inhibited the levels of proinflammatory cytokines and down-regulated the expression of HMGB1/LOX-1 in mouse models of *A. fumigatus* keratitis

**Figure 4:**
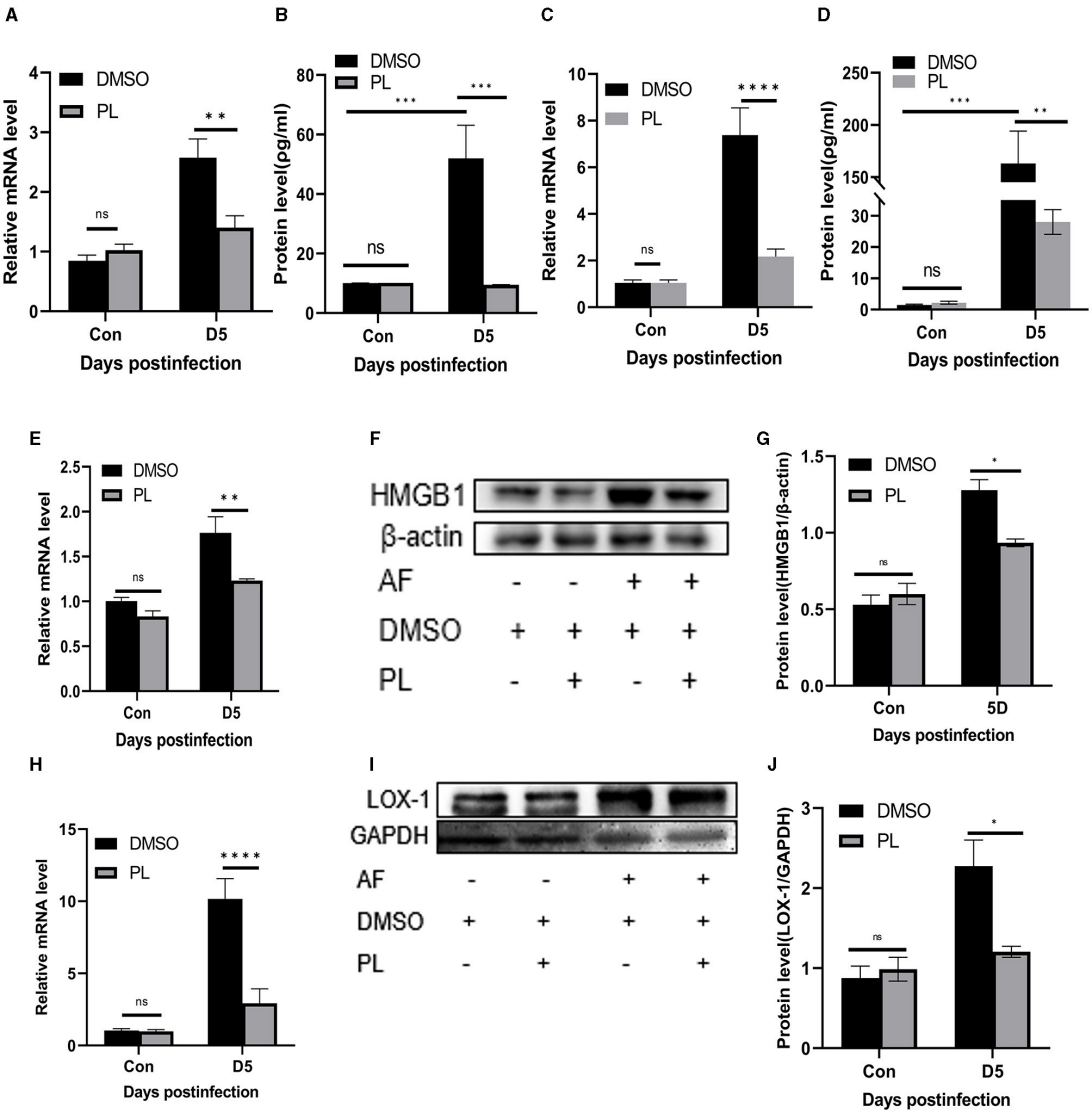
PL inhibited the inflammatory progression of *A. fumigatus* keratitis in mice. Compared with DMSO treatment group, the mRNA and protein levels of TNF-α **(A, B)** and IL-6 **(C, D)** in the cornea of PL treatment group were significantly decreased, and the expression of HMGB1/LOX-1 **(E–J)** was significantly down-regulated. All data were displayed as mean ± SEM (*n* = 6/group, ns, no significance; *P < 0.05, **P < 0.01, ***P < 0.001, ****P < 0.0001. Con, control, corneas in control group were not infected with *A. fumigatus*; D, days p.i.).

In addition to conducting *in vitro* experiments, we also conducted an assessment of the anti-inflammatory effect of PL on mouse corneas. Treatment with PL resulted in a significant down-regulation of proinflammatory cytokines TNF-α (Figures 4A, B) and IL-6 (Figures 4C, D) at both mRNA and protein levels compared to the DMSO treatment group at 5 days p.i. Furthermore, we investigated whether the mechanism underlying PL's efficacy in treating fungal keratitis is associated with HMGB1/LOX-1 by examining changes in mRNA and protein expression levels in the cornea. Our findings revealed a significant decrease in HMGB1/LOX-1 expression within the cornea of the PL treatment group compared to the DMSO treatment group (Figures 4E–J).

### 3.5 PL exerted an anti-inflammatory effect by inhibiting HMGB1/LOX-1 signaling pathway

**Figure 5:**
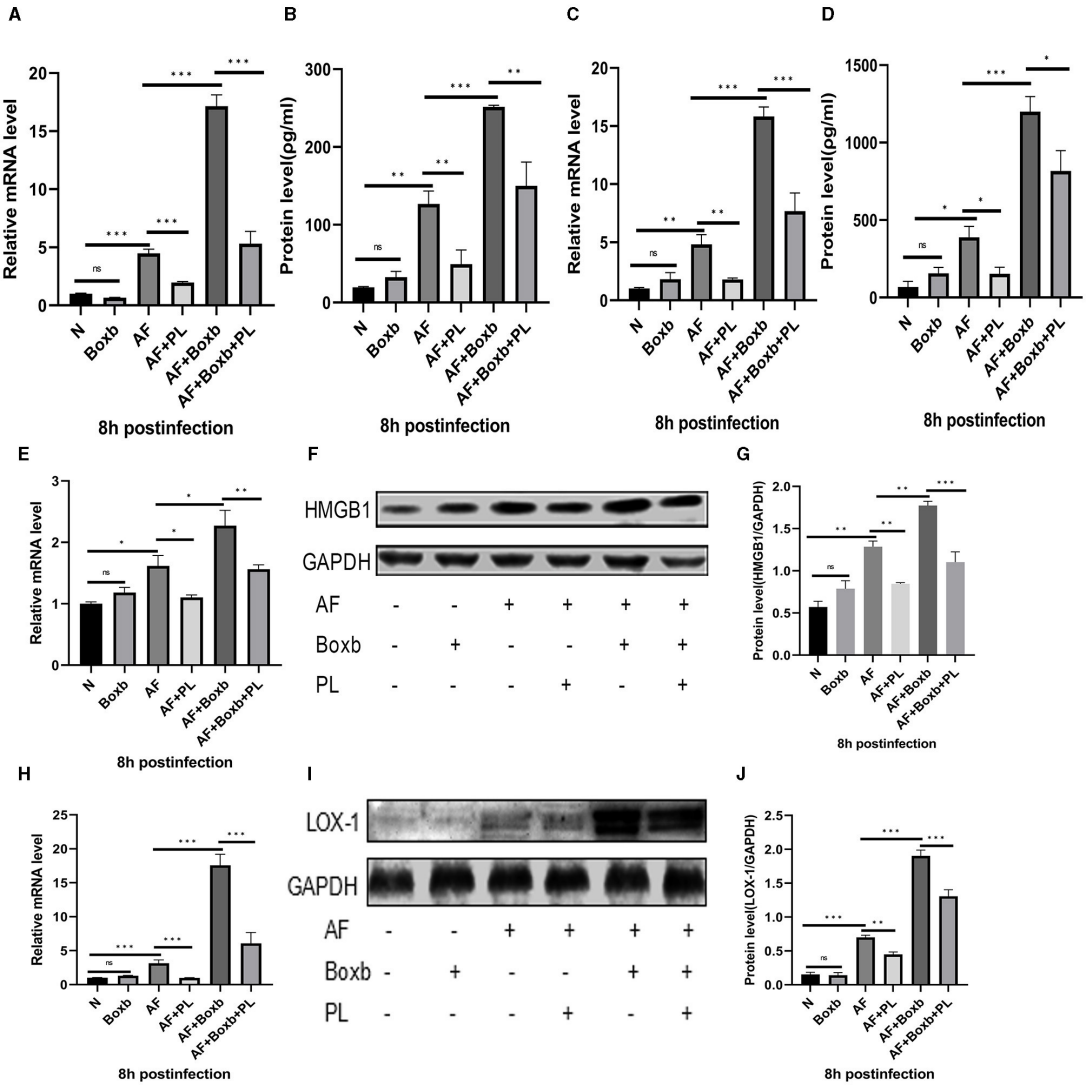
The inhibitory effect of PL on the inflammatory response was mediated through the inhibition of the HMGB1/LOX-1 pathway. Pre-treatment with Boxb significantly enhanced the mRNA and protein expression levels of HMGB1 **(E–G)**, LOX-1 **(H–J)**, TNF-α **(A, B)**, and IL-1β **(C, D)**. Furthermore, treatment with PL effectively attenuated the inflammatory amplification induced by Boxb (*n* = 6/group). All data were displayed as mean ± SEM (ns, not significant; *P < 0.05, **P < 0.01, ***P < 0.001. *N*, normal peritoneal macrophages; AF, *A. fumigatus*).

In order to further elucidate the mechanism of action of PL treatment on *A. fumigatus* keratitis, we employed pre-treatment with Boxb in mouse peritoneal macrophages. The results demonstrated that following *A. fumigatus* infection, PL treatment significantly reduced the protein levels of HMGB1 and LOX-1. Boxb stimulation increased the expression of HMGB1 and LOX-1 in cells; however, PL treatment could notably decrease Boxb-induced high expression of HMGB1 and LOX-1 (Figures 5E–J). Similar results were observed for TNF-α (Figures 5A, B) and IL-1β (Figures 5C, D). These findings suggested that PL could down-regulate LOX-1 expression by inhibiting HMGB1 activity, subsequently reducing IL-1β and TNF-α levels and exerting an anti-inflammatory effect.

### 3.6 The antifungal efficacy of PL *in vitro*

**Figure 6:**
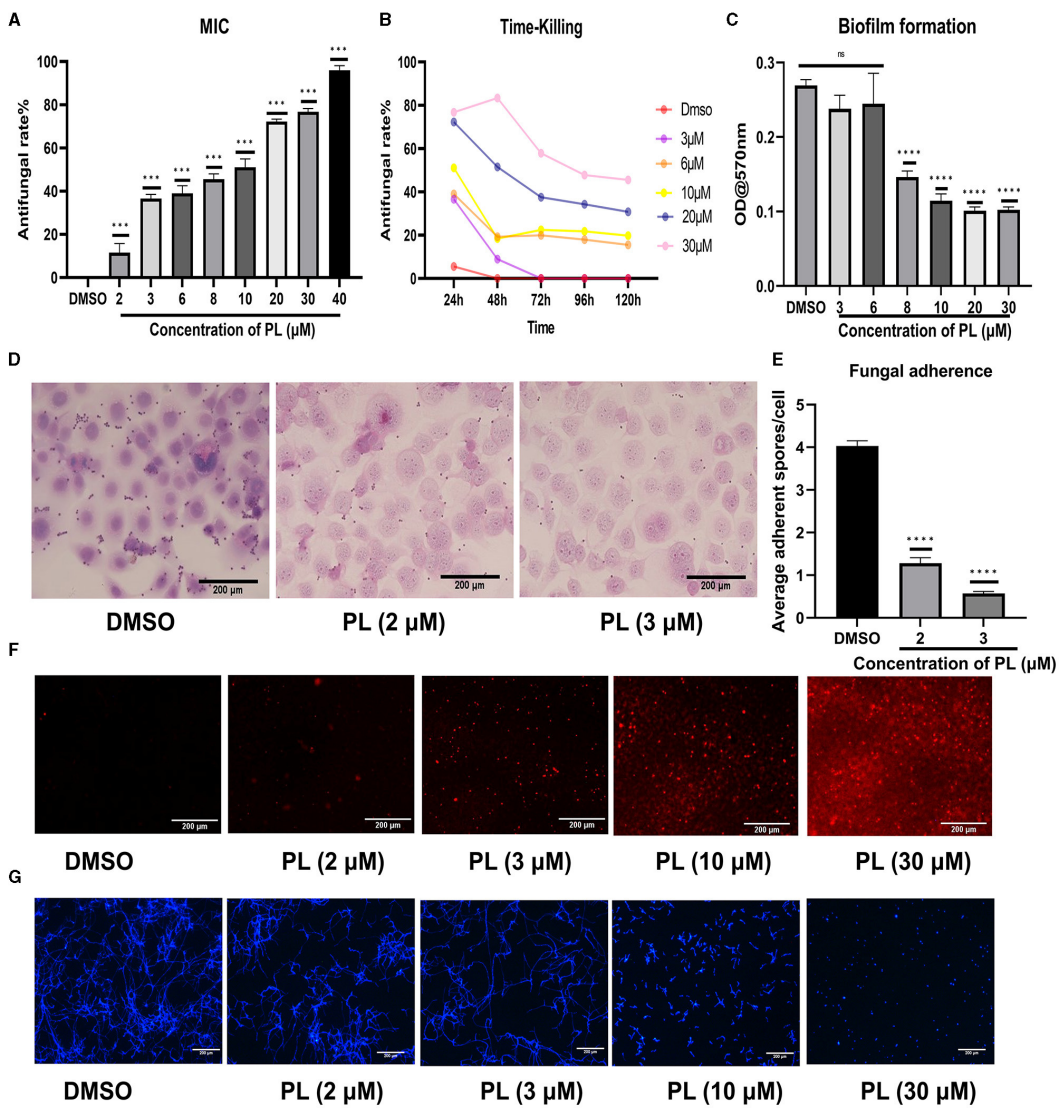
PL exhibited remarkable antifungal efficacy. The inhibitory effect of PL on *A. fumigatus* was assessed at different concentrations within 24 h **(A)**, and the change in inhibitory rate was monitored over a period of 120 h **(B)**. Crystal violet chromogenic assay confirmed the inhibitory effect of PL on biofilm formation **(C)**. PL inhibited spore adhesion *in vitro* (**D, E**; Magnification, 200×, bar: 200 μm). The antifungal effects of PL treatment at various concentrations were evaluated through PI staining **(F)** and calcofluor white staining **(G)** (magnification, 100×, bar: 200 μm). All data were displayed as mean ± SEM (ns, no significance; ^***^P < 0.001, ^****^P < 0.0001).

When PL was cocultured with conidia for 24 h, the growth of fungi was concentration-dependently inhibited from 1.25 μM (Figure 6A). It was observed that the inhibitory rate of PL at 3 μM could reach 37%, and 30 μM PL inhibited fungal growth by 77%. Although the inhibitory effect of PL decreased over time, even at 120 h, a concentration of 30 μM still exhibited effectiveness of 45% (Figure 6B). Our study demonstrated that PL could inhibit fungal biofilm formation, concentrations ≥ 8 μM were found to significantly impede biofilm development (Figure 6C). Furthermore, we confirmed that PL exhibited a significant reduction in spore adhesion to HCECs compared to DMSO treatment, with statistical significance observed at concentrations of 2 and 3 μM (Figures 6D, E). PL also exhibited its ability to disrupt the cell membrane and cell wall of *A. fumigatus*. PI staining images revealed a positive correlation between the fluorescence intensity of mycelia and PL concentration, indicating that PL caused slight damage to the integrity of the fungal membrane starting from 2 μM, with a pronounced damaging effect observed at 30 μM (Figure 6F). Calcium fluoride white fluorescence staining demonstrated that treatment with PL at concentrations ≥ 10 μM resulted in obviously thinner and shorter mycelial morphology in a concentration-dependent manner (Figure 6G). These findings underscored the remarkable antifungal activity possessed by PL.

### 3.7 Both PL and NATA attenuated *A. fumigatus* keratitis and had synergistic effects

**Figure 7:**
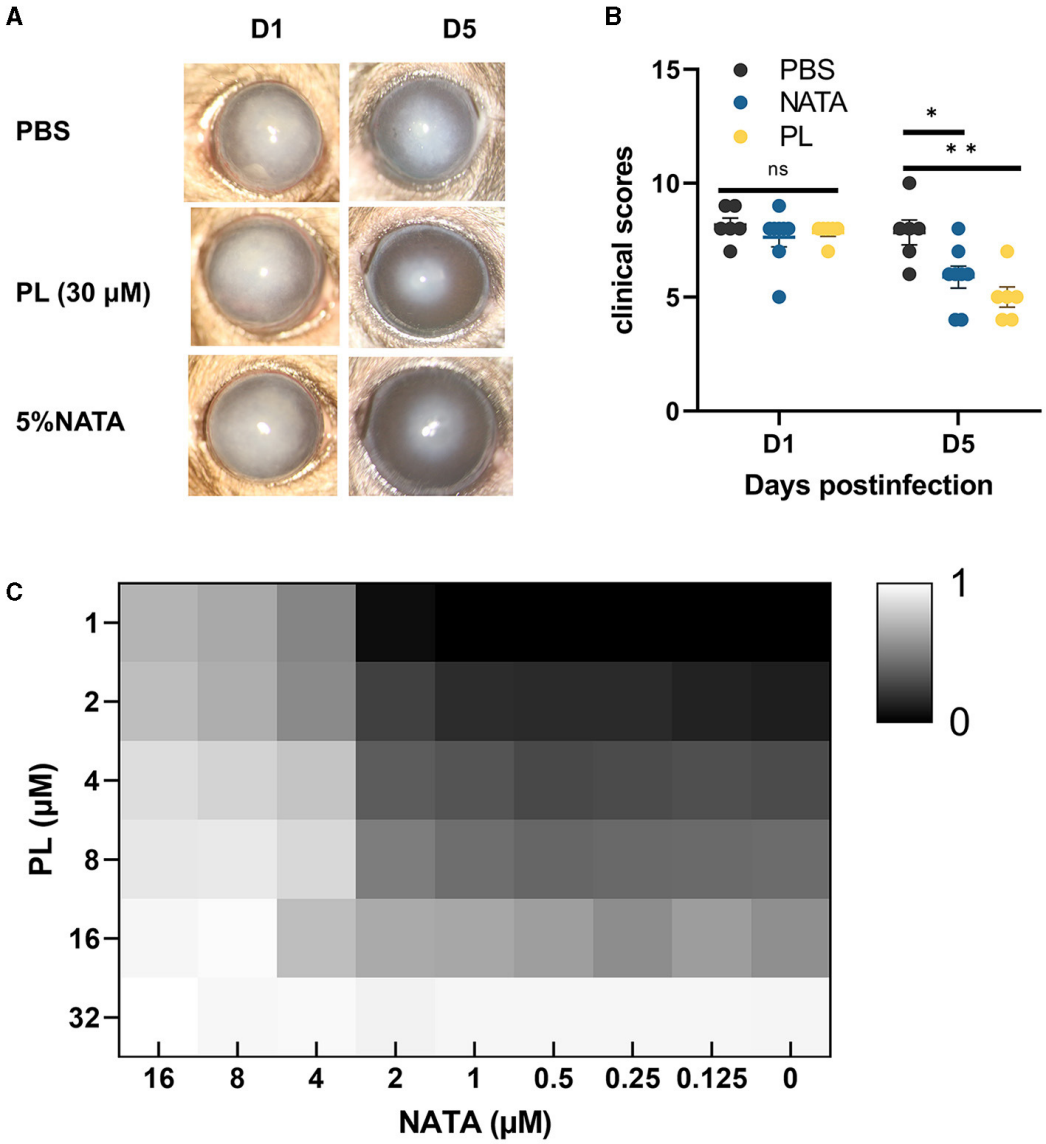
Comparison of efficacy and synergistic effect between PL and NATA. Corneal changed at 1 and 5 days p.i. after treatment with PBS, NATA or PL (**A**, *n* = 6/group) and analysis of differences between the three groups **(B)**. The synergistic effect of different concentrations of NATA (0–16 μM) and PL (1–32 μM) treated for 24 h was analyzed by checkerboard method, and the inhibition rate was calculated after reading OD **(C)**. All data were displayed as mean ± SEM (ns, no significance; *P < 0.05. D, days p.i.). **P < 0.01.

In order to compare the effects of PL and NATA on *A. fumigatus* keratitis, we treated mice with 30 μM PL, 5% NATA, and PBS. At 5 days p.i., significant reductions of corneal opacity area were observed in both the PL and NATA treatment groups, accompanied by alleviated edema and improved visibility of the iris (Figure 7A). The differences were statistically significant (Figure 7B). Additionally, synergy experiments were conducted using the checkerboard method (Figure 7C). Our findings demonstrated that 16 μM NATA and 32 μM PL can individually achieve MIC_70_. When used in combination, the concentrations of PL and NATA at MIC_70_ were both 4 μM. Therefore, the PL-NATA combination had a FICI value of 0.375 indicating a synergistic effect between them. The results from using either PL or NATA alone or in combination are presented in Table 2.

**Table 2:** FICI values for PL-NATA combination.

***A. fumigatus***							
**Compounds**		**Single MIC**		**Combined MIC**		**FICI**	**Result**
**A**	**B**	**A (**μ**M)**	**B (**μ**M)**	**A (**μ**M)**	**B (**μ**M)**		
NATA	PL	16	32	4	4	0.375	Synergistic

## 4 Discussion

The incidence of fungal keratitis is progressively increasing over the years (Oliveira dos Santos et al., 2020); 5% natamycin is considered the “gold standard” for treating fungal keratitis. Nevertheless, due to the poor dispersibility and low permeability of natamycin, 25% of cases still progress to corneal perforation following treatment (Hoffman et al., 2022).

The major active component of various plants, including *Plumbago indica L*., is PL, which has been demonstrated to possess a broad spectrum of biological and pharmacological activities. *In vitro* studies have shown that low doses of PL are non-toxic or exhibit low toxicity toward normal cutin cells and lens epithelial cells (Oh et al., 2017; Zhang et al., 2021). Furthermore, the safety of intravitreal injection of high doses of PL has been confirmed by previous research (Chen et al., 2018). These findings align with our study results and provide evidence for the safety of PL in *A. fumigatus* keratitis.

It is well-established that following fungal infection of the cornea, hyphae invade the corneal stroma and induce aggregation and infiltration of inflammatory cells. The growth of hyphae in the corneal stroma, along with protease secretion from neutrophil primary granules, lead to degradation of the corneal stroma, resulting in loss of corneal transparency (Ratitong and Pearlman, 2021). Through clinical scoring, plate counting, HE staining, and immunofluorescence, we observed that treatment with PL significantly ameliorated corneal edema, reduced ulcer area, and inhibited fungal growth and inflammatory cell infiltration, thereby improving corneal transparency, and the therapeutic efficacy of NATA in treating *A. fumigatus* keratitis is consistent with that of PL. Furthermore, previous studies have shown that PL effectively downregulates IL-1β expression in a mouse model of osteoarthritis (Wenhao Zheng et al., 2017), as well as inhibits TNF-α and IL-6 levels in a rat model of chronic periodontitis (Zheng et al., 2017), which aligns with our observations. We observed a significant inhibitory effect of PL on proinflammatory cytokines IL-1β, IL-6, and TNF-α expression in peritoneal macrophages while increasing anti-inflammatory cytokine IL-10 expression. These results suggest that PL exerts its anti-inflammatory effects by altering levels of inflammatory factors in *A. fumigatus* keratitis. In addition, neutrophils constitute over 90% of the infiltrating inflammatory cells in fungal keratitis (Ratitong and Pearlman, 2021), and previous studies have demonstrated that neutrophils are the primary source of IL-1β during infection (Peiró et al., 2018; Sun et al., 2018). The findings of our study demonstrate that PL effectively attenuated neutrophil infiltration in *A. fumigatus* keratitis. This observation is consistent with the fact that PL reduces neutrophil infiltration in liver tissue during fulminant liver failure (Wang et al., 2016). Therefore, it can be further inferred that the down-regulation of IL-1β levels by PL may also be attributed to its capacity for reducing neutrophil recruitment. In addition, PL had been reported to have anti-inflammatory effects in clinical trials, for example in allergic and COVID-19/uterine corpus endometrial carcinoma patients (Kohli et al., 2011; Li et al., 2021). Collectively, these results suggest that PL could potentially enhance the prognosis of fungal keratitis by inhibiting fungal growth and mitigating corneal inflammation in mice.

The extracellular form of HMGB1, known as damage-associated molecular pattern (DAMP), exhibits cytokine and chemotactic activity and plays a proinflammatory role in various disease models (Kang et al., 2014; Tang et al., 2023). Numerous studies have demonstrated the effectiveness of targeting HMGB1 as an anti-inflammatory strategy, showing that inhibiting HMGB1 can significantly reduce inflammatory responses in myocarditis, sepsis, periodontitis, and other diseases by down-regulating inflammatory cytokines such as IL-1β, TNF-α, and IL-6 (Yang H. et al., 2019; Jiang et al., 2021; Luo et al., 2022; Yang et al., 2022). Previous research on *Pseudomonas aeruginosa* keratitis has validated the potential of targeting HMGB1 as an anti-inflammatory approach (Ekanayaka et al., 2018; Hazlett et al., 2021). Meanwhile, PL has been shown to effectively inhibit HMGB1 in different disease models, including cholestatic liver injury, hepatic ischemic re-injury, and sepsis (Zhang Z. et al., 2016; Zaki et al., 2018; Pan et al., 2022). This research prompted us to conduct related experiments on corneal and peritoneal macrophages in mice. We observed that PL effectively suppressed the expression of HMGB1 both *in vitro* and *in vivo*, leading to a significant down-regulation of TNF-α, IL-6, and IL-1β. These results confirmed that PL may play an anti-inflammatory role in *A. fumigatus* keratitis by inhibiting HMGB1. It is well-known that LOX-1 plays a role in increasing the expression of proinflammatory cytokines during *A. fumigatus* keratitis (Che et al., 2018; Sun et al., 2019). It has been reported that HMGB1 is positively associated with changes in LOX-1, IL-1β, and TNF-α in murine models of COPD and acute lung injury (Liu et al., 2018; Xu et al., 2020). However, to date, no study has demonstrated a significant correlation between PL and LOX-1. To further investigate the mechanism of action of PL in fungal keratitis, relevant experiments were conducted. We observed that PL treatment not only suppressed the expression of HMGB1 but also attenuated the expression of LOX-1, while concurrently downregulating the levels of inflammatory factors such as IL-1β. Additionally, LOX-1 has the ability to recruit neutrophils (Yin et al., 2022). We have confirmed the efficacy of PL in reducing neutrophil infiltration, indicating a potential correlation between decreased LOX-1 expression and the inhibition of neutrophil infiltration by PL. We further validated the anti-inflammatory mechanism of PL by using an HMGB1 agonist, Boxb. Our findings demonstrated that PL significantly attenuated the enhanced expression of HMGB1 and LOX-1 mediated by Boxb, as well as the elevation of inflammatory cytokines induced by HMGB1. These results suggest that the anti-inflammatory effect of PL is achieved through the inhibition of HMGB1/LOX-1. In addition, excessive intracellular accumulation of ROS can impact multiple cellular signaling pathways, thereby triggering various pathological processes, including inflammatory responses (Zhang J. et al., 2016; Yang G.-G. et al., 2019). It is well-established that down-regulating the expression of HMGB1 and LOX-1 can inhibit intracellular ROS levels (Gao et al., 2016; Zhou et al., 2021), and in models of liver injury and upper respiratory tract inflammation, ROS can stimulate the release of HMGB1, which subsequently triggers an inflammatory immune response and generates more ROS (Bystrom et al., 2017; Min et al., 2017). Therefore, we hypothesize that there might be a cycling of ROS-HMGB1 during the inflammatory response. Previous studies have demonstrated the capacity of PL to attenuate intracellular ROS levels (Chen et al., 2019; Zhang et al., 2020). To validate this, we conducted pertinent *in vitro* experiments and substantiated the reduction of intracellular ROS levels by PL. Consequently, it can be further inferred that the anti-inflammatory mechanism of PL may also involve inhibiting ROS production and disrupting the ROS-HMGB1 cycle.

The initial stage of fungal infection involves the adhesion of spore to the corneal surface (Niu et al., 2020). We conducted experiments on this aspect and discovered that PL effectively inhibited the adhesion of *A. fumigatus* spores. Previous research has demonstrated that PL can disrupt the structural integrity of bacterial and fungal cell membranes and walls, leading to increased permeability and subsequent cellular demise (Periasamy et al., 2019; Shang et al., 2019; Qian et al., 2022; Wang et al., 2022). A similar effect of PL on *A. fumigatus* keratitis was also confirmed in our study, where the destruction of the cell membrane and cell wall was enhanced in a concentration-dependent manner. Additionally, during the growth of *A. fumigatus*, the biofilm produced exhibits protective and adhesive properties, leading to increased drug resistance (Di Somma et al., 2020). However, previous reports have indicated that PL can inhibit biofilm formation (Qian et al., 2022; Wang et al., 2022). Therefore, we conducted experiments related to biofilm formation and found that PL significantly inhibited its development. More significantly, PL demonstrated notable antifungal effects against clinical isolates of *Candida albicans* (Hassan et al., 2016), indicating the potential for clinical application in treating fungal keratitis. These findings suggest that PL possesses multifaceted antifungal effects, which are concentration-dependent, and exhibits promising therapeutic efficacy against *A. fumigatus* keratitis.

Additionally, the potential synergistic interaction between PL and NATA was investigated. Previous studies have reported a synergistic effect of PL with tetracyclines (Xu et al., 2022). By employing the checkerboard method, our results showed that PL could exhibit synergy effect with NATA against *A. fumigatus*, thereby reducing the required drug concentrations and improving the prognosis of *A. fumigatus* keratitis. These findings suggest that PL has the potential to serve as a potent adjunctive therapeutic agent, highlighting its significant prospects for treating *A. fumigatus* keratitis.

The potential of PL in alleviating *A. fumigatus* keratitis lies in its ability to inhibit the growth of *A. fumigatus*, suppress inflammation by regulating the HMGB1/LOX-1 signaling pathway and inhibiting ROS production. Therefore, PL holds promise for the remission and treatment of *A. fumigatus* keratitis.

## Data availability statement

The raw data supporting the conclusions of this article will be made available by the authors, without undue reservation.

## Ethics statement

Ethical approval was not required for the studies on humans in accordance with the local legislation and institutional requirements because only commercially available established cell lines were used. The animal study was approved by the Ethics Committee at Qingdao University's Affiliated Hospital. The study was conducted in accordance with the local legislation and institutional requirements.

## Author contributions

FC: Conceptualization, Methodology, Writing – original draft. LG: Supervision, Writing – review & editing. JL: Data curation, Validation, Writing – review & editing. GL: Data curation, Software, Writing – review & editing. QW: Formal analysis, Supervision, Writing – review & editing. LZ: Data curation, Software, Writing – review & editing. MC: Investigation, Methodology, Writing – review & editing. QX: Funding acquisition, Software, Writing – review & editing. GZ: Funding acquisition, Supervision, Visualization, Writing – review & editing. CL: Funding acquisition, Project administration, Resources, Writing – review & editing.
